# Probing the Hall anomaly and electronic structure in kagome metal RbV_3_Sb_5_ under hydrostatic pressure

**DOI:** 10.1080/14686996.2026.2675214

**Published:** 2026-05-29

**Authors:** Tsz Fung Poon, Zheyu Wang, Lingfei Wang, Ying Kit Tsui, Zikai Zhou, Wenyan Wang, Chun Wai Tsang, Alexandre Pourret, Gabriel Seyfarth, Georg Knebel, Swee K. Goh

**Affiliations:** a Department of Physics, The Chinese University of Hong Kong, Shatin, Hong Kong, China; b State Key Laboratory of Quantum Information Technologies and Materials, The Chinese University of Hong Kong, Shatin, Hong Kong, China; c Quantum Science Center of Guangdong-Hong Kong-Macao Greater Bay Area, Shenzhen, China; d Univ. Grenoble Alpes, CEA, Grenoble-INP, IRIG, Pheliqs, Grenoble, France; e Univ. Grenoble Alpes, CNRS, Univ. Toulouse, INSA-T, LNCMI-EMFL, Grenoble, France

**Keywords:** Kagome superconductor, Fermiology, high pressure, quantum oscillations, non-monotonic Hall effect, mobility spectrum analysis

## Abstract

Kagome metals AV${\;}_{3}$Sb${\;}_{5}$ (A = K, Rb, Cs) are renowned for their intricate electronic band structures, providing a rich platform for investigating topological states and electronic correlations. Within the AV${\;}_{3}$Sb${\;}_{5}$ family, the detailed electronic structures of CsV${\;}_{3}$Sb${\;}_{5}$ and KV${\;}_{3}$Sb${\;}_{5}$ have been well established, both in their charge-density-wave (CDW) phase or in the pristine metallic phase. Yet, the electronic structure of RbV${\;}_{3}$Sb${\;}_{5}$ remains under-explored. In this manuscript, we present a detailed study of the electronic structure of RbV${\;}_{3}$Sb${\;}_{5}$ revealed by Shubnikov–de Haas oscillation in both the CDW phase (9 kbar), and where the CDW phase is just fully suppressed (22 kbar). The greatly simplified Fast Fourier transform spectrum at 22 kbar implies the absence of Fermi surface reconstruction caused by the CDW order, and the observation of enhanced quasi-particle effective masses near the CDW boundary indicates enhanced quantum fluctuations. Furthermore, the mobilities of charge carriers from the CDW phase to the metallic pristine phase are studied using mobility spectrum analysis (MSA). Our MSA results reveal that high-mobility carriers (≈10,000 cm ${\;}^{2}$/Vs) coexist with the non-monotonic Hall effect near-zero field at 9 kbar. As the pressure increases to 22 kbar and 30 kbar, this non-monotonic feature is suppressed, concomitant with the disappearance of the high-mobility carriers. By summarizing the MSA results across the AV${\;}_{3}$Sb${\;}_{5}$ family, we observed that the superconducting behavior appears to be positively correlated with carrier mobility and number. This correlation suggests that high-mobility carriers may play a crucial role in the underlying superconducting pairing mechanism.

## IMPACT STATEMENT

This manuscript investigates the pressure evolution of the electronic structure of RbV_3_Sb_5_ using Shubnikov–de Haas quantum oscillations and mobility spectrum analysis, offering new insight into the physics of pressure-tuned kagome metal.

## GRAPHICAL ABSTRACT

**Figure:**
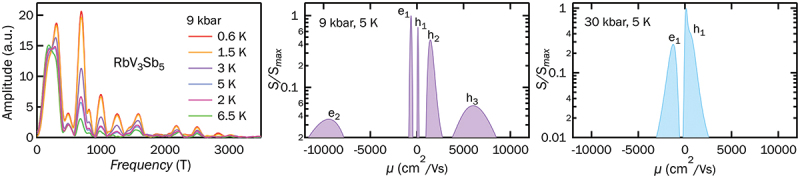

## 1. Introduction

Kagome metals AV${\;}_{3}$Sb${\;}_{5}$ (A = K, Rb, Cs) feature an electronic band structure characterized by van Hove singularities, Dirac points, and flat bands, establishing them as prime platforms for investigating the interplay between topologically nontrivial states and electronic correlations [1–6]. These materials exhibit a rich variety of electronic instabilities, including charge density wave (CDW) order, nematicity, and superconductivity (SC) [1,2,7–25], making them a central focus of contemporary condensed matter research

The complex Fermiology of the AV${\;}_{3}$Sb${\;}_{5}$ family underpins the rich phenomena observed in these materials. For example, the anisotropy and the temperature dependence of the upper critical field in CsV${\;}_{3}$Sb${\;}_{5}$ have been attributed to the anisotropy of the Fermi velocity originating from van Hove singularities [26]. To advance our understanding of the electronic structure of the AV${\;}_{3}$Sb${\;}_{5}$ family, techniques such as quantum oscillation (QO) measurements [17,21,27–32] and angle-resolved photoemission spectroscopy (ARPES) [32–37] have been utilized to probe their Fermi surfaces. However, the formation of a CDW introduces a new periodicity and Brillouin zone folding, resulting in Fermi surface reconstruction and complicating the analysis of the underlying electronic structure. A promising route to access the pristine metallic Fermi surface is to suppress the CDW order via hydrostatic pressure. QO studies on CsV${\;}_{3}$Sb${\;}_{5}$ [38] and KV${\;}_{3}$Sb${\;}_{5}$ [39] have shown that the Fermi surface changes drastically upon applying hydrostatic pressure across the CDW boundary, and the quasi-particle effective masses are enhanced near the CDW boundary. However, the pressure evolution of the Fermi surface of RbV${\;}_{3}$Sb${\;}_{5}$ remains unexplored. Thus, a detailed study of the Fermi surface evolution in RbV${\;}_{3}$Sb${\;}_{5}$ is crucial for a comprehensive understanding of the AV${\;}_{3}$Sb${\;}_{5}$ family.

Another intriguing phenomenon associated with the CDW phase in AV${\;}_{3}$Sb${\;}_{5}$ is the non-monotonic Hall effect close to zero field. Since its discovery [16,18,40–42], the origin of this effect has raised considerable debate, as it cannot be explained by conventional Hall effect or a two-band models. Proposed mechanisms include spontaneous time-reversal symmetry breaking via loop currents [23,43,44], Berry curvature effects [45,46], and proximity to van Hove singularities [47]. More recently, the near zero field non-monotonic Hall effect in CsV${\;}_{3}$Sb${\;}_{5}$ was attributed to the emergence of extremely high-mobility carriers associated with small Fermi pockets, as revealed by mobility spectrum analysis (MSA) [42]. While detailed investigations of carrier mobility using MSA have been conducted on KV${\;}_{3}$Sb${\;}_{5}$ [39] and CsV${\;}_{3}$Sb${\;}_{5}$ [42], such studies have not yet been performed for RbV${\;}_{3}$Sb${\;}_{5}$.

In this work, we present detailed magnetotransport measurements on RbV${\;}_{3}$Sb${\;}_{5}$ under hydrostatic pressure up to 29 T, targeting three representative pressure regimes in the temperature-pressure phase diagram: within the CDW phase (9 kbar), at the CDW phase boundary (22 kbar), and in the pristine metallic phase (30 kbar). Shubnikov – de Haas oscillation analysis reveals a pronounced simplification of the Fermi surface as the CDW order is fully suppressed, as evidenced by a reduced set of FFT frequencies in the QO spectrum, and the general enhancement of the quasi-particle effective mass, consistent with results from other AV${\;}_{3}$Sb${\;}_{5}$ compounds [27–30,32,38,39,41,48,49]. Furthermore, our MSA shows that the number of distinct carrier types decreases dramatically, accompanied by the suppression of the near zero field non-monotonic Hall effect upon applying pressure. The non-monotonic Hall effect near zero field vanishes in the pristine phase, where the MSA spectrum reduces to just two peaks, aligning with two-band transport behavior. The existing MSA results of AV${\;}_{3}$Sb${\;}_{5}$ at ambient or low pressures will be summarized, enabling an evaluation of the A-site dependence. Our discovery offers important insights into the nature of the near zero field non-monotonic Hall effect in Kagome metals.

## 2. Methods

Single crystals of RbV${\;}_{3}$Sb${\;}_{5}$ were synthesized via the self-flux method, as described in Ref [16]. A thin flake was mechanically exfoliated from the bulk crystal and transferred onto pre-patterned electrodes on a diamond anvil. The flake thickness was determined to be 360 nm using a dual-beam focused ion beam system (Thermo Scientific Scios 2, Thermo Fisher Scientific, United States). High-pressure environments were achieved using the device-integrated diamond anvil cell (DIDAC) technique [38,50–53]. Standard four-point electrical transport measurements were performed in a Physical Property Measurement System (PPMS): Quantum Design, United States below 14 T. High-field magnetotransport data were collected at the Laboratoire National des Champs Magnétiques Intenses (LNCMI) in Grenoble using a ${\;}^{3}$ He cryostat at fields up to 29 T, where quantum oscillation signals were detected by a standard four-point lock-in technique using a Stanford Research SR830 lock-in amplifier: Stanford Research System, United States. To extract carrier mobilities, mobility spectrum analysis (MSA) [39,42,54–60] was applied to the transverse magnetoresistance (MR) and Hall resistivity data simultaneously, with the magnetic field aligned along the c-axis. Specifically, the maximum entropy approach proposed by Ref. [57] was utilized. The detailed mathematical framework of this procedure is available in Ref. [60].

## 3. Results and discussions

**Figure 1.**
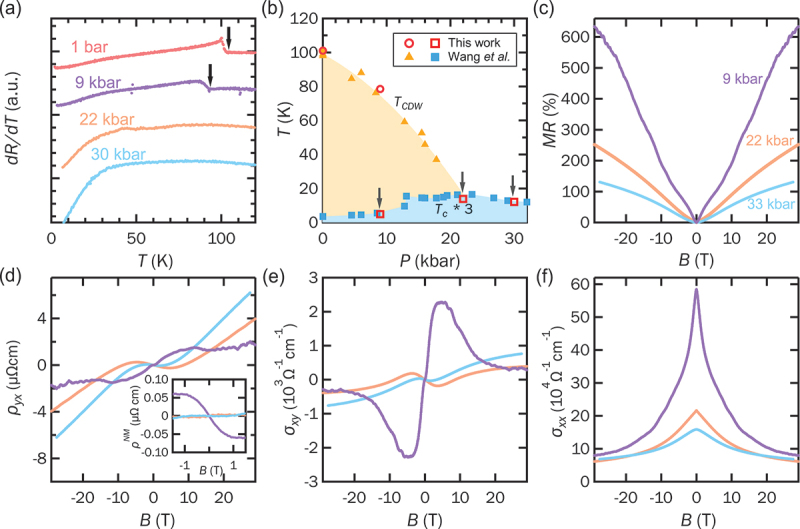
(a) The temperature dependence of the derivative of resistance of RbV_3_Sb_5_ at different pressures, the anomaly indicated by the black arrow corresponds to the CDW transition, the curves are vertically shifted for clarity. (b) Temperature-pressure phase diagram of RbV${\;}_{3}$Sb${\;}_{5}$, the closed symbols are adopted from Ref. [61]. The open symbols are data measured in this work. Both $T_{\mathrm{CDW}}$ and $T_{c}$ are consistent with previous study. (c) Magnetoresistance of RbV${\;}_{3}$Sb${\;}_{5}$ at 5 K under different pressures. (d) Hall resistivity $\rho_{\mathrm{yx}}$ of RbV_3_Sb_5_ at 5 K under different pressures. The inset shows the non-monotonic component of $\rho_{\mathrm{yx}}$ in the low-field region (within ±1 t). Hall conductivity and magnetoconductivity are presented in (e) and (f), respectively.

Figure 1(a) displays the temperature derivative of the resistance for RbV${\;}_{3}$Sb${\;}_{5}$ under various pressures. The anomalies in the derivative, marked by the black arrows, correspond to the CDW transition. Above 22 kbar, the $T_{\mathrm{CDW}}$ is fully suppressed and no longer detectable in the resistance derivative. Figure 1(b) presents the temperature-pressure phase diagram of RbV${\;}_{3}$Sb${\;}_{5}$ from Wang et al. [61]. Both $T_{\mathrm{CDW}}$ and the superconducting critical temperature ($T_{c}$) observed in our pressurized RbV${\;}_{3}$Sb${\;}_{5}$ samples are consistent with their findings. The arrows in Figure 1(b) indicate the key pressure points examined in this study: within the CDW phase at 9 kbar, at the CDW phase boundary at 22 kbar, and the pristine phase at 30 kbar. To investigate the pressure dependence of magnetotransport in RbV${\;}_{3}$Sb${\;}_{5}$, we applied magnetic fields up to 29 T along the crystallographic c-axis. Figure 1(c) summarizes the magnetoresistance (MR) at 5 K as a function of field. At 9 kbar, the MR (=$\frac{R(B)-R(0)}{R(0)}$) reaches up to 600% at 29 T, indicative of the high quality of our samples. The MR at 9 kbar displays clear wiggles at high fields, attributable to Shubnikov – de Haas (SdH) oscillations. The detailed SdH oscillations study will be presented in the next section. Across all pressures, the MR exhibits non-quadratic behavior. Figure 1(d) shows the Hall resistivity ($\rho_{\mathrm{yx}}$) at different pressures. At 9 kbar, $\rho_{\mathrm{yx}}$ exhibits clear non-monotonic, ‘S-shaped’ behavior near zero field (within ±1 T), which is a common feature across the AV${\;}_{3}$Sb${\;}_{5}$ family [16,18,40–42]. This anomaly was immediately attributed to anomalous Hall effect (AHE) when it was first discovered [16,18,40,41]. However, a recent mobility spectrum analysis (MSA) study on CsV${\;}_{3}$Sb${\;}_{5}$ shows that this Hall anomaly can also be explained by the presence of high mobility carriers within an ordinary multi-carrier framework [42]. Since our MSA result in the later part also points toward a similar conclusion, we have adopted ‘non-monotonic Hall behavior’ in this manuscript to describe the near zero-field Hall anomaly. As the pressure increases to the CDW boundary (22 kbar) and beyond (30 kbar), the Hall response evolves toward a more conventional two-band behavior. The inset of Figure 1(d) highlights the non-monotonic component of $\rho_{\mathrm{yx}}$ at various pressures, obtained by subtracting a linear low-field background. Notably, this non-monotonic Hall effect is prominent only within the CDW phase and vanishes outside it. Using the measured longitudinal and Hall resistivities, we calculated the Hall conductivity ($\sigma_{\mathrm{xy}}$) and magnetoconductivity ($\sigma_{\mathrm{xx}}$) via $\sigma_{\mathrm{xx}/xy}=\frac{\rho_{\mathrm{xx}/yx}}{\rho_{xx}^{2}+\rho_{yx}^{2}}$, as shown in Figure 1(e,f), respectively.

**Figure 2.**
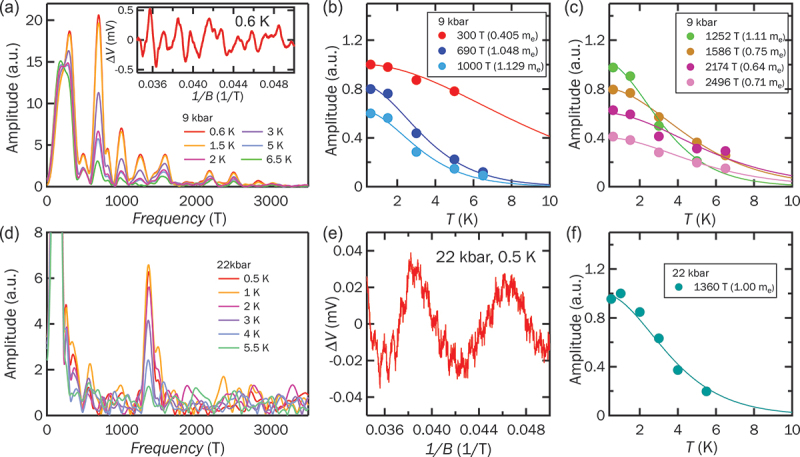
FFT spectrum of RbV${\;}_{3}$Sb${\;}_{5}$ for the oscillatory data between 20 and 29 T at different temperatures at (a) 9 kbar, and (d) 22 kbar. The inset of (a) shows the oscillatory signals after the removal of the background at 0.6 K, 9 kbar. The oscillatory signals at 22 kbar are shown in (e). The temperature dependence of the quantum oscillation amplitudes at (b)–(c) 9 kbar, and (f) 22 kbar. The solid lines are the fitting result of the thermal damping factor in LK theory.

Next, we examine the evolution of the Fermi surface upon applying hydrostatic pressure by QO studies. The QO signals can be obtained by subtracting the MR background with a high-order polynomial. The resultant oscillatory signals at 9 kbar and 0.6 K are presented in the inset of Figure 2(a). The main panel of Figure 2(a) displays the Fast Fourier Transform (FFT) spectrum for magnetic fields between 20 and 29 T at 9 kbar, revealing numerous FFT frequencies below 3000 T, indicative of a complex Fermi surface. The temperature dependence of the FFT amplitudes is plotted in Figure 2(b,c). The effective mass ($m^{*}$) of the quasi-particle can be found by fitting the temperature dependence of the FFT amplitude to the thermal damping factor $R_{T}$ in Lifshitz – Kosevich (LK) theory,

$$R_{T}=\frac{14.693m^{*}T/B}{\sinh(14.693m^{*}T/B)},$$

where B is the applied magnetic field. The fitting results are plotted as solid lines in Figure 2(b,c). Notably, the $m^{*}$ values in RbV${\;}_{3}$Sb${\;}_{5}$ at 9 kbar are generally larger than those observed at ambient pressure [16,27,41]. Compared with the ambient pressure SdH oscillations results [16], the effective mass increases from 0.12 to 0.41 $m_{e}$ for the 300 T peak, from 0.44 to 1.0 $m_{e}$ for the 690 T peak, from 0.47 to 0.75 $m_{e}$ for the 1586 T peak, and from 0.20 to 0.64 $m_{e}$ for the large 2174 T peak. When the pressure is increased to 22 kbar, at which the CDW phase is just fully suppressed, the QO signal and the corresponding FFT spectrum are observed in Figure 2(e,d), respectively. Compared to the 9 kbar data, the strong peaks below 1000 T are no longer observable, leaving only a relatively weak peak near 1300 T. Since the signal-to-noise ratio calculated for this peak increases from 17.8 at 9 kbar to 19.6 at 22 kbar, it suggests that the simplification of the FFT spectrum is intrinsic, rather than a loss of signal-to-noise ratio. Unfortunately, the signal-to-noise ratio of 30 kbar data is too low, thus we do not include it for comparison. As determined from the LK fit shown in Figure 2(f), the $m^{*}$ of the surviving frequency remains close to $1m_{e}$, close to its low-pressure value. Similar to its sister compounds, the intricate multi-peak structure observed between 1000 and 3000 T at lower pressures can be attributed to Fermi surface reconstruction driven by the CDW transition [32,38]. As the pressure exceeds the CDW phase boundary, this reconstruction is suppressed, resulting in a significantly simplified FFT spectrum. Furthermore, a substantial enhancement of the quasi-particle $m^{*}$ near the CDW phase boundary would render certain FFT peaks hardly detectable. This explains why only one peak remains visible at 22 kbar despite the high signal-to-noise ratio. Indeed, the significant increase in $m^{*}$ is already observed at 9 kbar compared with that of ambient pressure, which implies that the quasi-particle $m^{*}$ tends to increase when the pressure approaches the CDW boundary. Such an enhancement suggests the quantum fluctuations strengthened near the phase boundary, consistent with recent superconducting critical current experiments [53,61]. Alternatively, this mass enhancement could, in principle, be attributed to the Fermi energy being tuned toward a van Hove singularity under applied pressure. However, this scenario typically applies only to a specific band, and it cannot account for the general enhancement of $m^{*}$ across multiple orbits as suggested by our data. Therefore, our observations more naturally point toward the influence of quantum fluctuations. In summary, the spectrum of FFT frequencies, corresponding to the extremal orbits of the Fermi surface, undergo a dramatic simplification without the CDW order. The observed general enhancement of the quasi-particle $m^{*}$ provides insights into potential quantum criticality. The changes in Fermi surface topology are expected to profoundly influence the nature of the charge carriers, as discussed in the following section.

**Figure 3.**
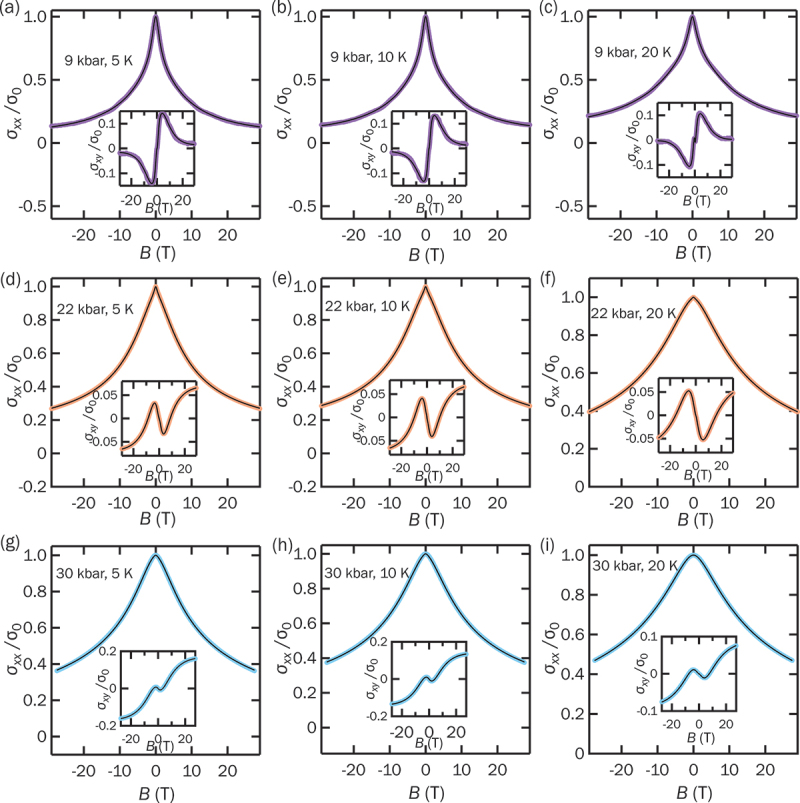
Normalized magnetoconductivity ($\sigma_{\mathrm{xx}}/\sigma_{0}$) of RbV${\;}_{3}$Sb${\;}_{5}$ at (a) 5 K, (b) 10 K, and (c) 20 K at 9 kbar. $\sigma_{\mathrm{xx}}/\sigma_{0}$ at (d) 5 K, (e) 10 K, and (f) 20 K at 22 kbar. $\sigma_{\mathrm{xx}}/\sigma_{0}$ at (g) 5 K, (h) 10 K, and (i) 20 K at 30 kbar. The normalized Hall conductivity $\sigma_{\mathrm{xy}}/\sigma_{0}$ are included in the insets. The black solid lines represent the MSA fitting results.

To extract carrier mobilities, we simultaneously analyzed the transverse magnetoresistance and Hall resistivity using Mobility Spectrum Analysis (MSA). Within the Maximum Entropy Mobility Spectrum Analysis framework [57], we can express the magnetoconductivity normalized to the zero field magnetoconductivity value ($\sigma_{\mathrm{xx}}/\sigma_{0}$) and the normalized Hall conductivity ($\sigma_{\mathrm{xy}}/\sigma_{0}$) as:

$$\frac{\sigma_{\mathrm{xx}}}{\sigma_{0}}=\sum_{i}\frac{p_{i}}{1+{\left(\mu_{i}B\right)}^{2}}\;\mathrm{and}$$

$$\frac{\sigma_{\mathrm{xy}}}{\sigma_{0}}=\sum_{i}\frac{p_{i}\mu_{i}B}{1+{\left(\mu_{i}B\right)}^{2}},$$

where $\mu_{i}$ is the mobility, B represents the field, and $p_{i}$ is the probability distribution. The index i go through different mobilities, and the mobility spectrum can be obtained by fitting $p_{i}$. The mobility spectrum is obtained by the relation: $s(\mu_{i})/s(\mu_{\max})=p_{i}/p_{\max}$, where $p_{\max}$ is the maximum probability. The detailed fitting procedure can be found in Ref [60]. This approach allows for an unbiased interpretation of magnetotransport data, as it does not require prior assumptions regarding the number of carrier species. Figure 3(a–c) display $\sigma_{\mathrm{xx}}/\sigma_{0}$ at 9 kbar for temperatures ranging from 5 K to 20 K. The insets show the corresponding $\sigma_{\mathrm{xy}}/\sigma_{0}$. The black solid lines represent the MSA fits, which show excellent agreement with the experimental data, confirming the validity of the model. At 9 kbar, a distinct non-monotonic behavior in $\sigma_{\mathrm{xy}}$ is observed, which is successfully captured by the MSA fitting. We further examined the transport behavior at higher pressures. Figure 3(d–f) present $\sigma_{\mathrm{xx}}/\sigma_{0}$ and $\sigma_{\mathrm{xy}}/\sigma_{0}$ at 22 kbar. At this pressure, the non-monotonic behavior disappears. Finally, at 30 kbar, where the pristine metallic phase is restored, the low-field non-monotonicity remains absent. In this regime, $\sigma_{\mathrm{xy}}$ exhibits behavior characteristic of a standard two-band metallic system.

**Figure 4.**
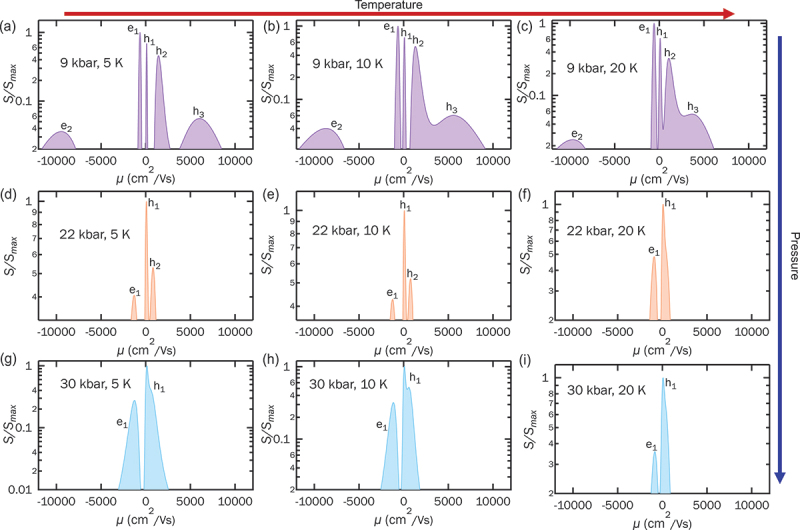
MSA spectra at (a) 5 K, (b) 10 K, and (c) 20 K at 9 kbar, five charge carrier types are extracted below 20 K. MSA spectra at (d) 5 K, (e) 10 K, and (f) 20 K at 22 kbar, three charge carrier types are extracted below 10 K, but only two are extracted at 20 K. MSA spectra at (g) 5 K, (h) 10 K, and (i) 20 K at 30 kbar, two charge carrier types are extracted below 20 K.

Leveraging these accurate MSA fits, we generate the MSA spectra to quantify the carrier mobilities across different pressure regimes. We first focus on the carrier dynamics of RbV${\;}_{3}$ Sb${\;}_{5}$ inside the CDW phase. Figure 4(a) shows the MSA spectrum at 5 K, where five distinct peaks are resolved: two electron carriers with mobilities of approximately 650 ($e_{1}$) and 10,000 ($e_{2}$) cm ${\;}^{2}$/Vs, and three hole carriers at around 110 ($h_{1}$), 1,430 ($h_{2}$), and 6,000 ($h_{3}$) cm ${\;}^{2}$/Vs. As the temperature increases to 10 K and 20 K (Figure 4b,c), all five peaks remain detectable, but the carrier mobilities decrease and the peaks begin to merge toward lower mobilities. Notably, the hole carrier $h_{3}$ that is well-separated from $h_{2}$ at 5 K, starts to merge with $h_{2}$ at 10 K, and at 20 K, $h_{2}$ and $h_{3}$ are almost completely overlapped. Upon increasing the pressure to 22 kbar, corresponding to the CDW phase boundary, the MSA spectrum at 5 K (Figure 4d) reveals only three peaks: one electron carrier ($e_{1}$) at 1,300 cm ${\;}^{2}$/Vs and two hole carriers at 70 and 810 cm ${\;}^{2}$/Vs. Compared to the low-pressure case, the high-mobility carriers are strongly suppressed under pressure, leaving only carriers with lower mobility. At higher temperatures, the MSA spectrum at 20 K (Figure 4f) shows that the two hole peaks ($h_{1}$ and $h_{2}$) merge, resulting in a simplified two-band scenario with one electron and one hole carrier, consistent with the two-band behavior observed in $\rho_{\mathrm{yx}}$ at 22 kbar (Figure 1d). At 30 kbar, in the pristine phase, the MSA spectrum at 5 K (Figure 4g) resolves only two peaks: one low mobility electron and one hole carrier. This two-band behavior persists up to 20 K, as shown in Figure 4(h,i). Previous studies of CsV${\;}_{3}$Sb${\;}_{5}$ [42] have attributed the non-monotonic Hall effect to the presence of high-mobility carriers. While introducing local impurities via irradiation has been shown to suppress high mobility carriers and diminish the non-monotonic Hall effect, we achieve a suppression of the non-monotonic behavior through hydrostatic pressure. Furthermore, increasing both temperature and pressure reduces carrier mobility, causing the high mobility peaks to merge with the low mobility ones in the mobility spectrum. This consistent evolution suggests that the non-monotonic Hall effect in the CDW phase of RbV${\;}_{3}$Sb${\;}_{5}$ is intimately linked to the presence of high-mobility carriers, which exhibit extreme sensitivity to external tuning via pressure and temperature.

**Figure 5.**
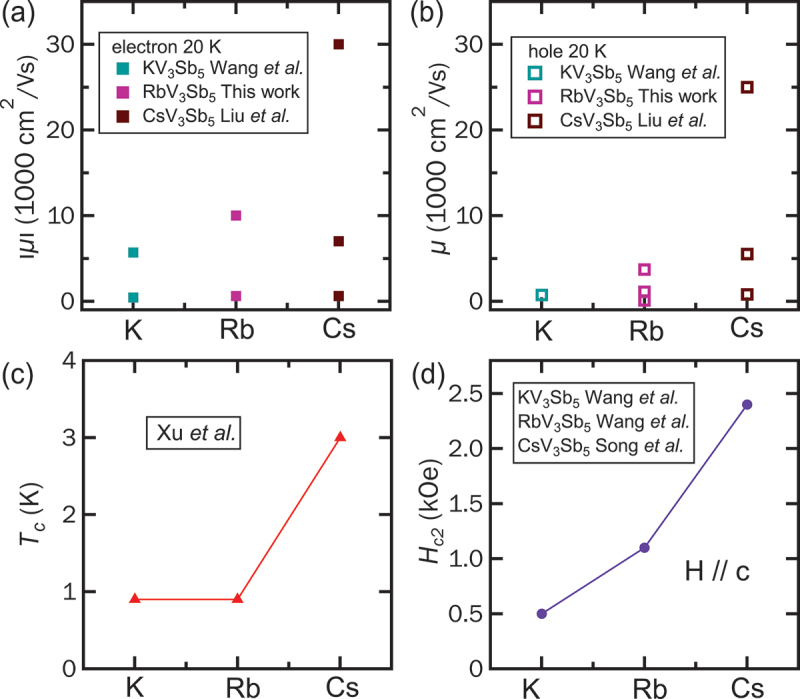
(a) The electron mobilities and (b) hole mobilities at 20 K for different members in AV${\;}_{3}$Sb${\;}_{5}$ family. Data from KV${\;}_{3}$Sb${\;}_{5}$ and CsV${\;}_{3}$Sb${\;}_{5}$ are taken from Refs. [39] and [42]. respectively. (c) Superconducting critical temperature $T_{c}$ taken from Ref. [62] and (d) the out-of-plane upper critical field $H_{c2}$ at the zero-temperature limit, summarized from Refs. [20,63,64] for different members in AV${\;}_{3}$Sb${\;}_{5}$ family.

To broaden our perspective on charge carrier mobility across the AV${\;}_{3}$Sb${\;}_{5}$ family, we compare the MSA results for K-, Rb-, and Cs-based compounds. Figure 5(a,b) summarize the electron and hole mobilities of all three members. Since the non-monotonic Hall effect is observed only in the CDW region, we use mobility data obtained under low pressure or at ambient pressure at 20 K. Specifically, for KV${\;}_{3}$Sb${\;}_{5}$, data were collected at 3 kbar from Wang et al. [39]; for CsV${\;}_{3}$Sb${\;}_{5}$, ambient pressure data were taken from Liu et al. [42]. The residual resistance ratio (RRR) of CsV${\;}_{3}$Sb${\;}_{5}$ [65] and RbV${\;}_{3}$Sb${\;}_{5}$ is found to be around 80, while that of KV${\;}_{3}$Sb${\;}_{5}$ [17] is approximately 60. The comparable RRR values indicated that all samples are of similar quality; this is crucial for a valid comparison, as impurities have been shown to suppress the high mobility carrier and the non-monotonic Hall effect [42]. Among the AV${\;}_{3}$Sb${\;}_{5}$ family, CsV${\;}_{3}$Sb${\;}_{5}$ stands out with the highest carrier mobilities for both electrons and holes (up to ≈ 30,000 cm ${\;}^{2}$/Vs) and the largest number of carriers (three types each of electrons and holes) in the CDW phase. In contrast, RbV${\;}_{3}$Sb${\;}_{5}$ has noticeably reduced carrier mobilities (most below 10,000 cm ${\;}^{2}$/Vs) and one fewer electron carrier compared to CsV${\;}_{3}$Sb${\;}_{5}$. KV${\;}_{3}$Sb${\;}_{5}$ exhibits the lowest carrier mobilities and the fewest carrier types-two electron and one hole. Superconducting properties also vary significantly across the AV${\;}_{3}$Sb${\;}_{5}$ family. Figure 5(c) presents the superconducting transition temperature $T_{c}$ for each compound [62], and Figure 5(d) summarizes the out-of-plane upper critical field $H_{c2}$ at zero temperature [20,63,64] in bulk samples. Both KV${\;}_{3}$Sb${\;}_{5}$ and RbV${\;}_{3}$Sb${\;}_{5}$ exhibit $T_{c}\approx 0.9$ K, while CsV${\;}_{3}$Sb${\;}_{5}$ shows a substantially higher $T_{c}$ of ≈3 K. Despite similar $T_{c}$ values, the out-of-plane $H_{c2}$ of RbV${\;}_{3}$Sb${\;}_{5}$ (≈1100 Oe) is roughly twice that of KV${\;}_{3}$Sb${\;}_{5}$. Thus, the superconductivity of RbV${\;}_{3}$Sb${\;}_{5}$ appears more robust than KV${\;}_{3}$Sb${\;}_{5}$ even with comparable $T_{c}$. Overall, our MSA results reveal a clear dependence of superconducting properties on the A-site element. The strength of superconductivity appears to have a correlation with carrier mobility and carrier number: CsV${\;}_{3}$Sb${\;}_{5}$ with the highest mobility and greatest number of carriers, while KV${\;}_{3}$Sb${\;}_{5}$ has the fewest carrier types and lowest mobilities, corresponding to the weakest superconductivity. These findings suggest that high-mobility carriers may play a crucial role in mediating superconductivity and the robustness of the pairing state in AV${\;}_{3}$Sb${\;}_{5}$ kagome metals.

The origin of the variation in the number of carrier types across different members of AV${\;}_{3}$Sb${\;}_{5}$ can possibly be linked to their distinct nature of the CDW order. When the CDW order sets in, the 2×2×4 tri-hexagonal (TrH) – star of David (SoD) stacking in CsV${\;}_{3}$Sb${\;}_{5}$ introduces a longer periodicity along the c-axis than the 2×2×2 TrH – TrH stacking in RbV${\;}_{3}$Sb${\;}_{5}$ and KV${\;}_{3}$Sb${\;}_{5}$. Since the pristine Fermi surface is not completely cylindrical, the out-of-plane order is expected to reconstruct the Fermi surface by Brillouin zone folding along the $k_{z}$ direction. Thus, the longer c-axis period for CsV${\;}_{3}$Sb${\;}_{5}$ is expected to induce a more severe Fermi-surface reconstruction, potentially giving rise to more Fermi pockets than in RbV${\;}_{3}$Sb${\;}_{5}$ and KV${\;}_{3}$Sb${\;}_{5}$. This difference may explain why more carrier types are detected by MSA in CsV${\;}_{3}$Sb${\;}_{5}$. In other words, the difference in the number of carrier types can be traced to the difference in the CDW ordering vectors. Interestingly, a recent study also showed that the distinct CDW patterns have a profound impact on superconductivity [66]. Specifically, the SoD structure in CsV${\;}_{3}$Sb${\;}_{5}$ can preserve van Hove singularities near the Fermi level, which can promote s-wave SC with enhanced $T_{c}$ via bond-order fluctuations. Therefore, the different CDW patterns – hence the different ordering vectors – in members of the AV${\;}_{3}$Sb${\;}_{5}$ family may qualitatively link the $T_{c}$ enhancement to the number of carrier types detected in MSA. Finally, the observation that CsV${\;}_{3}$Sb${\;}_{5}$ has the highest mobility carriers than RbV${\;}_{3}$Sb${\;}_{5}$ and KV${\;}_{3}$b${\;}_{5}$ is interesting – Liu et al. [42] showed that these carriers are likely to originate from certain small pockets. Thus, it is natural to speculate that such small pockets arise from reconstruction by 2×2×4 ordering. Clarifying the microscopic origin of the high-mobility carriers, therefore, becomes an important direction for follow-up work.

## 4. Conclusion

Through the application of hydrostatic pressure, we systematically investigated the evolution of the Fermi surface in RbV${\;}_{3}$Sb${\;}_{5}$, achieving full suppression of the CDW phase at 22 kbar. Our results reveal a significant simplification of the Fermi surface, evidenced by the markedly cleaner FFT spectrum. This suppression of Fermi surface reconstruction upon the collapse of the CDW phase appears to be a universal feature within the AV${\;}_{3}$Sb${\;}_{5}$ family [38,39]. Furthermore, the observed enhancement of the quasi-particle effective mass as pressure approaches the CDW boundary points toward enhanced quantum fluctuations in this regime. Our mobility spectrum analysis (MSA) further identifies a high-mobility carrier (≈10,000 cm ${\;}^{2}$/Vs) that coexists with the non-monotonic Hall effect. Notably, the pressure-induced suppression of this non-monotonic feature is consistently accompanied by the disappearance of these high-mobility carriers. Systematic comparison of the MSA data across the AV${\;}_{3}$Sb${\;}_{5}$ family reveals that superconducting behavior appears to correlate positively with both the carrier number and the carrier mobilities. These findings offer valuable insights into the origin of superconductivity and its variation within the AV${\;}_{3}$Sb${\;}_{5}$ family, shedding light on the underlying pairing mechanism.
